# AI solutions for overcoming delays in telesurgery and telementoring to enhance surgical practice and education

**DOI:** 10.1007/s11701-024-02153-9

**Published:** 2024-11-11

**Authors:** Yang Li, Nicholas Raison, Sebastien Ourselin, Toktam Mahmoodi, Prokar Dasgupta, Alejandro Granados

**Affiliations:** 1 Surgical and Interventional Engineering, King’s College London, London, UK; 2 Department of Engineering, King’s College London, London, UK; 3 Department of Urology, Guy’s Hospital, London, UK

**Keywords:** Telesurgery, Telementoring, Surgical education, Artificial intelligence

## Abstract

Artificial intelligence (AI) has emerged as a transformative tool in surgery, particularly in telesurgery and telementoring. However, its potential to enhance data transmission efficiency and reliability in these fields remains unclear. While previous reviews have explored the general applications of telesurgery and telementoring in specific surgical contexts, this review uniquely focuses on AI models designed to optimise data transmission and mitigate delays. We conducted a comprehensive literature search on PubMed and IEEE Xplore for studies published in English between 2010 and 2023, focusing on AI-driven, surgery-related, telemedicine, and delay-related research. This review includes methodologies from journals, conferences, and symposiums. Our analysis identified a total of twelve AI studies that focus on optimising network resources, enhancing edge computing, and developing delay-robust predictive applications. Specifically, three studies addressed wireless network resource optimisation, two proposed low-latency control and transfer learning algorithms for edge computing, and seven developed delay-robust applications, five of which focused on motion data, with the remaining two addressing visual and haptic data. These advancements lay the foundation for a truly holistic and context-aware telesurgical experience, significantly transforming remote surgical practice and education. By mapping the current role of AI in addressing delay-related challenges, this review highlights the pressing need for collaborative research to drive the evolution of telesurgery and telementoring in modern robotic surgery.

## Introduction

Modern robotic surgery is undergoing a transformative revolution driven by the convergence of cutting-edge technologies such as artificial intelligence (AI), 5G/6G wireless communication, edge computing, and the internet of surgical things (IoST). These advancements are reshaping the way surgeries are performed, allowing for unprecedented precision, speed, and real-time decision-making. AI plays a pivotal role in enhancing robotic systems by enabling them to process vast amounts of data, recognise patterns, and make predictive decisions that improve surgical outcomes. 5G/6G wireless networks, with their ultra-low latency and high data transfer rates, facilitate real-time communication between surgeons and robotic systems, even over long distances. Meanwhile, edge computing brings computational power closer to the surgical site, reducing processing delays, saving communication resources, and ensuring rapid feedback. The IoST, which connects and orchestrates various medical devices and sensors in the operating room, enables seamless multimodal data sharing.

At the forefront of these advancements, telesurgery and telementoring are ushering in a new era of remote surgical care and mentorship, addressing a critical need for accessible specialist support. For example, the National Cardiothoracic Centre in Ghana reports that Africa has the lowest number of specialist surgeons globally, with only 0.5 specialists per 100,000 residents compared to 36.2 per 100,000 in Europe [1]. Broader clinical adoption of telesurgery and telementoring could alleviate such shortages, enhancing healthcare access in Africa and other underserved regions. Moreover, telesurgery and telementoring provide a cost-effective alternative to traditional mentorship models, eliminating travel expenses and reducing time off work for surgeons, making expert guidance more accessible and sustainable [2].

**Fig. 1:**
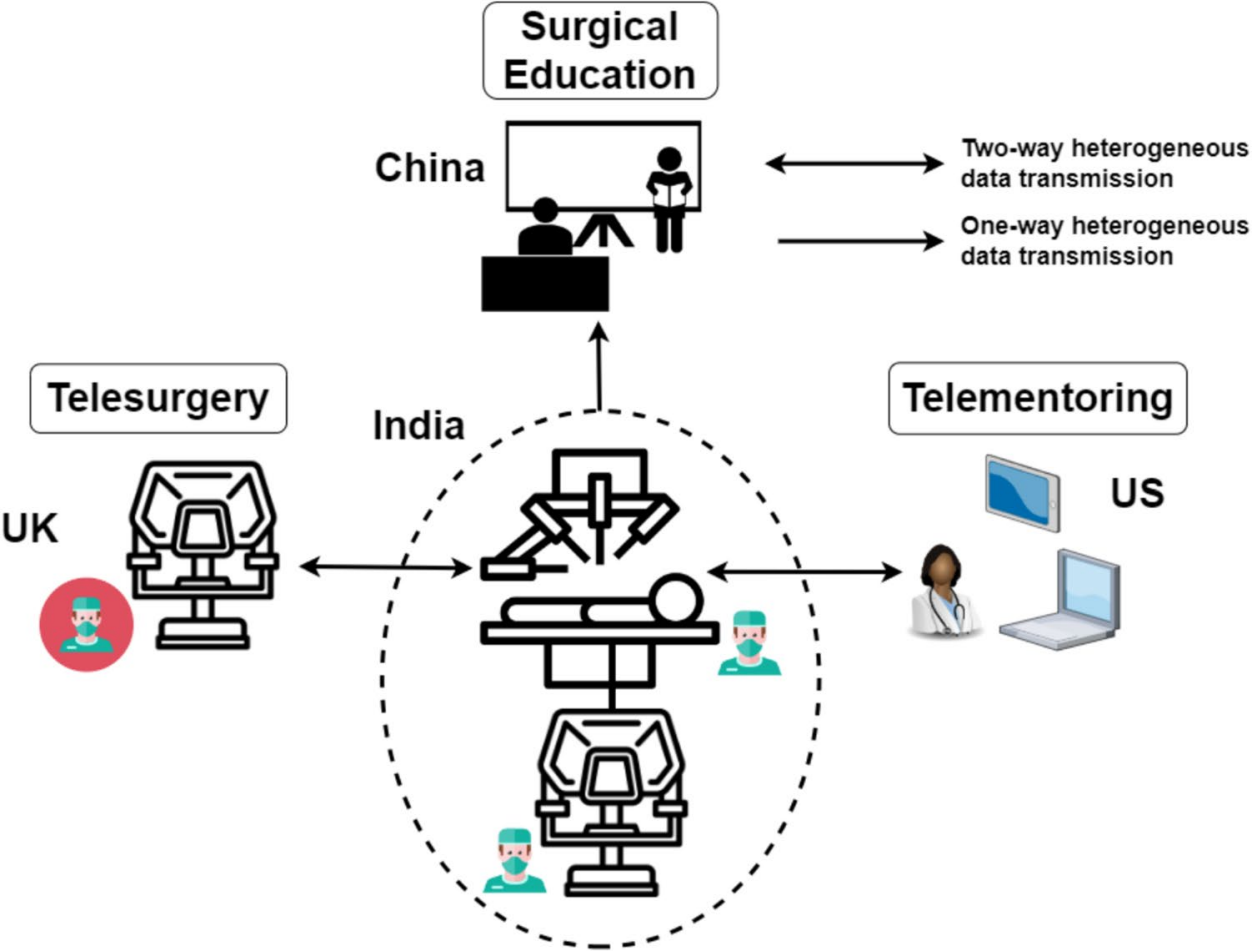
Telesurgery and telementoring for surgical practice and education. While a surgical team typically performs surgery and trains the next generation of surgeons locally (dashed circle line), expertise can be provided from other locations by controlling a robotic system remotely (telesurgery) or by augmenting the endoscopic view with audiovisual information (telementoring), or by a combination of both. Surgical trainees are also able to connect remotely in real time or retrospectively (surgical education)

The first transatlantic robotic surgical intervention was performed in 2001 using the percutaneous access of the kidney (PAKY) robot [3]. With telesurgery, physical distance no longer hinders the provision of life-saving interventions between surgical teams and patients [4]. Highly skilled surgeons can now perform intricate procedures by remotely controlling surgical instruments attached to robotic systems. For example, a surgeon based on the UK can operate on a patient in India, as shown in Fig. 1. Enabled by high-speed communication and advanced surgical robotics, this capability brings specialised surgical care to those in remote or underserved areas.

With benefits alike, telementoring enables expert surgeons to provide invaluable real-time guidance and mentorship to surgeons and other members of surgical teams located in operating theatres anywhere in the world [5]. During this process, trainees can also passively observe surgeries from various local and remote locations, increasing their clinical exposure and learning opportunities, as illustrated in Fig. 1. This dynamic exchange of knowledge and experience is facilitated by real-time communication channels, state-of-the-art user interfaces (UIs), and augmented/mixed reality (AR/MR) technologies [6], ensuring mentoring is timely, efficient, accurate, and safe.

Despite their benefits, the clinical applications of telesurgery and telementoring remain limited due to unique technical, regulatory, and ethical challenges that must be addressed to realise their full potential. Among the technical challenges, one of the primary hurdles is the need for ultra-low latency communication systems; even the slightest delay in data transmission can have serious consequences in a surgical setting [7]. Regardless of future improvements in processing times and network speeds, delays are unavoidable due to physical distance constraints and the inherent limitations of light propagation. Furthermore, the loss of crucial data, such as video, interventional imaging, and sensor data streams, can be catastrophic during a procedure [8].

It is recognised that increasing latency places additional strain on surgeons, reduces surgical performance and extends operating times [9]. Technical standards recommend a maximum delay of 10 ms for haptic-enabled telesurgery [10], while some studies suggest that the maximum acceptable delays for telesurgery without haptics could be up to 50 ms [11]. However, studies have reported successful procedures being performed with delays exceeding 100 ms [12]. As shown in Table 1, recent studies have shown that average network delays of wire-connected telesurgery vary between 4 and 10 ms over hundreds of kilometres. Several experiments have been conducted to test the feasibility of telesurgery on commercial networks with successful results [13, 14], yet with significant scope for improvement.

**Table 1:** Network round-trip delays under different experimental settings

Work	Device	Data	Tech	Distance (km)	Mean Delay (ms)
Ebihara et al. (2023) [14]	Riverfield Inc	Visual, Control	Bandwidth Allocation	250	8 (3–31)
Morohashi et al. (2022) [13]	Riverfield Inc	Visual, Control	Guaranteed-type line	150	4 (4–7)
			Best effort-type line	150	10 (9–13)
Zhang et al. (2022) [15]	MicroHand S	Visual, Control	Fixed-line DetNet	240	5 (5–6)
			5G DetNet	210	26 (20–57) / 27 (22–53)

While the delays shown in Table 1 are attributed to data transmission times, data processing delays also significantly impact the performance of telesurgery and telementoring systems. Morohashi et al. [13] examined glass-to-glass time, which is the total time for video or audio transmission from source to target, and reported delays of 92–95 ms, despite network delay is only around 4 ms. This indicates that processing delays can contribute substantially to the overall latency experienced in remote surgical applications.

It is noteworthy that the studies listed in Table 1 transmit only visual and robot control data in their experiments, without incorporating audio and haptic data. This is primarily because current surgical robots are not yet capable of providing robust haptic feedback. The addition of other data streams would further increase the data processing and transmission requirements, potentially exacerbating latency issues.

These challenges are further highlighted by the diverse range of data streams required for effective telesurgery and telementoring. The selection of data streams should align with the available technology and the specific needs of the surgical team and trainees. In telesurgery, potential sources of data are auditory, visual, control signals, and sometimes even tactile feedback [4]. Instrument kinematic and pressure data are essential to achieve tactile feedback. In telementoring, some argue that only auditory and visual data are needed [16]. However, others propose more interactive telementoring sessions where the trainee surgeon is at a remote console that mirrors the expert surgeon’s console [17]. For a truly immersive and interactive remote experience, there is a growing demand for more diverse data streams to help reconstruct the remote context [18].

Existing reviews have extensively explored their general concepts [19–23], applications to specific surgical specialities [24–33], advancements in video technology and intelligent control systems [6, 34, 35], surgeon and robotic training methodologies [36, 37], and even considerations regarding their carbon footprint [38]. Yet, routine clinical applications of telemedicine, especially in surgery, remain uncommon. AI offers unique potential to address many of the current challenges inherent in telesurgery and telementoring. Therefore, the aim of this paper is to provide a review of how AI can contribute to more efficient and robust transmission and utilisation of data, thereby enhancing remote surgical practice and education. By examining AI-driven solutions to technical challenges, particularly those related to latency and data integrity, we seek to highlight opportunities for facilitating broader and more immersive adoption of telesurgery and telementoring.

## Methods

A review of the literature was undertaken in accordance with the principles of the preferred reporting items for systematic reviews and meta-analyses (PRISMA) guidelines [39]. A single author performed a literature search according to the following inclusion criteria: journal, conference, and symposium papers written in English investigating the applications of AI in addressing delays and developing delay-robust applications.

**Table 2:** Keywords used for searching studies related to AI, surgery, teleoperation and delays

Topic	Keywords
AI	(“Artificial Intelligence” OR “Deep Learning” OR “Machine Learning” OR “Neural Network”) AND
Surgery	(“Surgery” OR “Surgical”) AND
Teleoperation	(“Teleoperated” OR “Tele-operated” OR “Telepresence” OR “Tele-presence” OR “Telemedicine” OR “Tele-medicine” OR “Telesurgery” OR “Tele-surgery” OR “Remote Surgery” OR “Telementoring” OR “Tele-mentoring” OR “Robotic” OR “Robot” OR “Teleproctor” OR “Tele-proctor” OR “Teleassessment” OR “ Tele-assessment”) AND
Delay	(“Delay” OR “Delays” OR “Latency” OR “Latencies”)

IEEE Xplore and PubMed were used to acquire original research articles published from 2010 until the end of October 2023. We stratified our search keywords into four groups: a) AI-related terms, b) surgery-related terms, c) teleoperation-related terms, and d) delay-related terms. The complete list of keywords is shown in Table 2. On IEEE Xplore, the search was limited to the abstract field, whereas on PubMed, it was limited to the title and abstract fields. However, delay-related keywords were searched on all data fields to include more relevant results.

From the selected literature, we extracted information including, but not limited to, the goal of the study (e.g., delay minimisation, developing delay-robust application), the type of experiment (*in vivo*, *ex vivo*, *in vitro*, *in silico*), the type or modalities of data used (e.g., video, audio, control signals), the AI techniques employed, and the implications in surgical practice and education.

**Fig. 2:**
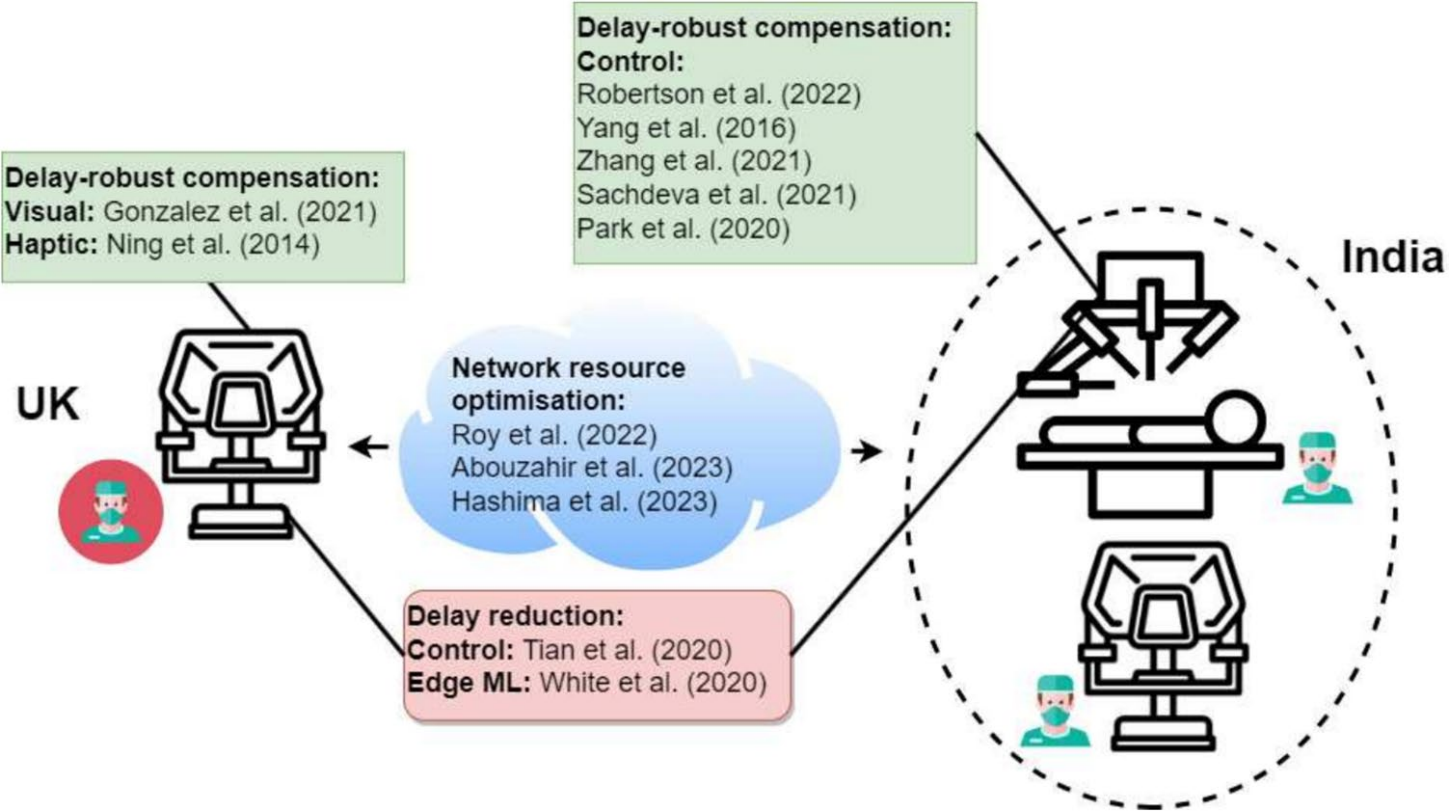
Diagram depicting the focuses of each study under the telesurgery scenario between the UK and India. The network resource optimisation studies are listed in the blue cloud. Other studies that are not directly about networking are listed according to their deployment location and solution type. The green squares contain studies on delay-robust compensation schemes. The delay reduction studies are included in the red rounded box. The black lines are linked to the location where they are deployed

## Results

Our search identified twelve studies in total, which can be stratified into three research directions (see Fig. 2). First, three studies propose AI models to optimise the networking resources available for efficient communication. Second, two studies present AI techniques aim at minimising delays resulting from local and remote data processing. Finally, seven studies acknowledge that delays are inevitable and propose AI methodologies that are robust to these delays. An overview of AI-powered techniques and other technical terms is included in Table 3. The identified key studies, along with details of their proposed models, are listed in Table 4.

**Table 3:** Glossary of technical innovations facilitating the delivery of telesurgery and telementoring

Name	Description
Network and Surgery	
5G	The characteristics of the 5G network are millisecond-level of end-to-end latency, and connection density of more than $10^6 devices/km^2$ [40]
6G	The characteristics of the 6G network are on-air latency of 10–100 $\mu$s, end-to-end latency <1 ms, reliability beyond 99.99%, and connection density of more than $10^7 devices/km^2$ [41]
Blockchain	A form of distributed technology that enables secure, transparent, and tamper-resistant record-keeping of transactions across a network of computers. It maintains trust between entities [41]
DetNet	Deterministic networking that enables the reliable delivery of critical and time-sensitive data [15]
E2E	End-to-End (e.g., mobile phone to another mobile phone) [42]
QoS	Optimise the use of network resources and provide a predictable and consistent level of service for different types of traffic [43]
QoE	Assesses how well users perceive and interact with a particular service or application [43]
Surgeme	Atomic surgical maneuvers [44]
AI	
cGAN	A type of generative model that can be used for content generation subject to a condition, such as segmentation labels of an image [45]
CNN	A type of artificial neural network that extracts important features from inputs such as images via convolutional layers that process data in small windows and via fully connected layers for end tasks such as classification [46]
Edge ML	Training and running AI models on edge devices such as mobile phones [47]
FL	A method of training AI models while keeping data local [43]
LSTM	A type of recurrent neural network capable of memorising previous events to estimate future outcomes [43]
Mask-RCNN	Based on CNN, designed for segmentation [44]
RBF NN	A type of artificial neural network that is used in many applications, such as modelling the stress characteristics of a surface and function approximation [48]
RBP	Learns the minimum resource required to meet a requirement [43]
Segmentation	Delineating the boundaries of objects observed in images [44]
Transfer learning	Update the last layers of pre-trained models at the edge of the network, reducing the training time [47]
U-net	A type of CNN, suitable for medical image segmentation [49]
YoloV3	A type of artificial neural network capable of detecting an object in an image [44]

**Network and others:** DetNet: Deterministic Networking; E2E: End-to-End; QoS: Quality of Service; QoE: Quality of Experience. **AI:** cGAN: conditional Generative Adversarial Network; CNN: Convolutional Neural Network; FL: Federated Learning; LSTM: Long–Short-Term Memory; NN: Neural Network; RBF: Radial Basis Function; RBP: Robust Banach-Picard; RCNN: Region-based Convolutional Neural Networks; YoloV3: You Only Look Once, Version 3

**Table 4:** Studies resulting from our search and eligibility criteria proposing AI techniques for addressing delays in telesurgery and telementoring

References	AI technique	Data	Validation	Surgery	Description
Network resource optimisation					
Roy et al. [42]	NN - learn the relationship between resources and the E2E QoE outcomes	Input: CPU and bandwidth usage; Output: E2E delays and throughput	In silico	N/A	Optimising network link and server resource
Abouzahir et al. [43]	LSTM and RBP - predict future channel gain based on previous channel gain	Input: current channel gain; Output: future channel gain	In silico	N/A	A predictive QoS allowing edge devices to adjust their power consumption intelligently under URLLC’s constraints
Hashima et al. [50]	AI softwarisation - learn the selection of ultra-fast optimal policy	Input: channel conditions, traffic demand, user mobility, etc.; Output: ultra-fast optimal policy	In silico	N/A	Intelligent policy selection with softwarisation
Delay reduction					
Tian et al. [46]	CNN - learn the robot control function	Input: robot joint information and end effector information; Output: control signal	In silico	N/A	Low-latency control algorithm
White et al. [47]	Edge ML - image classification on edge	Input: images (dogs and cats); Output: classification results	In silico	N/A	Transfer learning on edge for reduced processing delay
Delay-robust compensation					
Gonzalez et al. [44]	LSTM, YoloV3, Mask RCNN - learn the characteristics of high-level surgemes	Input: robot kinematic data; Output: surgeme label and parameter	In vitro	Peg transfer	Abstracting low-level image data into high-level command data
Ning et al. [48]	RBF NN - learn to synchronise visual and haptic information	Input: predicted coordinates of the remote robot agent; Output: force feedback time	In silico	N/A	Force feedback time prediction
Robertson et al. [51]	“Snap-Surface”and“Real-Track”	Input: 3D video and CT scan of the patient’s face; Output: co-registered images	Ex vivo	Bedside cranial intervention (human cadaveric heads)	A portable automatic stereotactic surgical robot
Yang et al. [52]	RBF NN - approximates non-linear control function	Input: master robot joint displacement, velocity, and acceleration; Output: slave robot joint information	In silico	N/A	Error constrained and delay-tolerant control
Zhang et al. [53]	LSTM - learn physiological tremor patterns	Input: previous tremor signal; Output: predicted tremor signal	In vitro	Vascular interventional surgery (phantom)	Physiological tremor prediction
Sachdeva et al. [45]	cGAN - learn to segment tooltips in images	Input: endoscopic camera view; Output: segmentation masks	In silico	mock gastrointestinal surgery	Detection of tooltip position
Park et al. [49]	U-net - learn to segment tooltips in images	Input: endoscopic camera view; Output: segmentation labels	Ex vivo	Needle insertion (porcine corneas)	Detection of tooltip and tissue position

cGAN: conditional Generative Adversarial Network; CNN: Convolutional Neural Network; LSTM: Long–Short-Term Memory; NN: Neural Network; RBF: Radial Basis Function; RBP: Robust Banach-Picard; RCNN: Region-based Convolutional Neural Networks; YoloV3: You Only Look Once, Version 3

### Network resource optimisation

Wireless networks offer greater flexibility in deployment, lower costs, and better availability compared to optical fibre transmission. The ease of setting up wireless infrastructure allows for rapid deployment without the need for extensive physical cabling, which is beneficial in emergency situations or temporary setups. In addition, wireless networks enable mobility, allowing surgical teams to operate from various locations without being tethered to fixed network points. In contrast, although optical fibre provides high bandwidth and more stable and reliable connections, its physical speed limit is constrained by a higher refractive index, resulting in slower propagation compared to wireless transmission [54]. Despite all the benefits, one of the primary challenges for wireless networks is resource allocation for applications. Consequently, two identified studies focus on optimising resource allocation in 5G wireless networks to meet the stringent demands of telesurgery and telementoring.

One of the primary challenges in resource allocation for these applications is balancing network and computing resources to meet specific user requirements. Key evaluation metrics include delay, throughput, jitter, and reliability—all of which directly affect the end-to-end (E2E) quality of experience (QoE). For instance, high latency or jitter can degrade the quality of streamed video and haptic feedback, crucial for surgical precision. In the context of telesurgery and telementoring, E2E QoE reflects the perceptual differences between remote and traditional surgical and mentoring experiences, impacting the surgeon’s performance, patient safety, and trainee’s learning efficacy.

Modelling the relationship between resource allocation and E2E QoE is complex due to its stochastic and non-linear nature. Without an accurate model, it is challenging to allocate the optimal amount of network and computing resources to applications. Deep learning (DL) offers a solution by handling such complexity effectively. In [42], DL is applied to learn the relationship between network resource allocation and E2E QoE outcomes. When a new service or application initiates and requests specific resources, the DL model can infer parameters like maximum allowable delay and minimum required throughput. This enables further optimisation of allocated resources to meet the desired E2E QoE. Following this DL-based approach, the authors [42] report achieving approximately 99.8% accuracy in modelling E2E QoE.

In another study [43], a federated learning (FL) approach is developed to predict quality of service (QoS) requirements and adjust power consumption on edge devices. FL is a learning paradigm that allows geographically dispersed clients to collaboratively train models locally while keeping data private, a critical feature for sensitive surgical data. This approach is particularly suitable for internet of things (IoT) networks, where centralised data processing is impractical due to bandwidth and privacy concerns. As energy-consuming services such as telesurgery and telementoring expand, optimising energy consumption becomes essential to prevent performance bottlenecks. Instead of maximising computing resources indiscriminately, adjusting power consumption on edge devices ensures they deliver just the right amount of performance under ultra-reliable and low-latency communication (URLLC) constraints. Both studies aim to allocate optimal resources within current 5G frameworks.

Other studies have investigated 6G for real-time interactions in telesurgery and telementoring. The dynamic and heterogeneous nature of 6G networks introduces new challenges—static AI solutions may not be compatible with all situations and devices due to varying applications, network conditions, and device capabilities. To address this, an intelligent policy selection solution utilising softwarisation is proposed [50]. Softwarisation involves the use of software-defined networking and network function virtualisation (NFV) to create flexible, programmable networks that can adapt in real time.

AI models in 6G networks may incorporate various AI techniques like supervised learning [42, 43], unsupervised learning, reinforcement learning, and optimisation methods such as linear programming and multi-objective optimisation. These models tackle network-related problems including traffic management, mobility prediction, resource allocation, and energy efficiency. Softwarisation emphasises the need for on-demand and adaptive AI models that can be dynamically deployed to network elements like access points (APs) and base stations (BSs).

In particular, the multi-armed bandit (MAB) framework is highlighted for its ability to quickly select optimal policies in real-time, ensuring ultra-fast decision-making. MAB models are well-suited for the dynamic and heterogeneous environments of 6G networks because they can adapt quickly to changing conditions without the need for extensive pre-training.

### Processing delay reduction

To address processing delays, enabling edge and mobile deployment of AI is crucial for faster and more effective data processing. Running algorithms on edge devices (those located close to the data source rather than in distant cloud servers) is gaining popularity as it offers greater flexibility and potential for enhanced real-time performance. By processing data locally, edge computing reduces the need to transmit large amounts of data over networks, thereby decreasing latency and improving responsiveness.

In particular, training and running AI models on edge devices can save significant amounts of time and reduce networking resource consumption [47]. Edge devices demonstrate faster response times compared to using cloud services. Furthermore, by utilising transfer learning, pre-trained models can be quickly fine-tuned and deployed on edge devices, reducing the time and computational resources required to train models from scratch.

Another area for improving performance is optimising the control algorithms of surgical robots, especially those with multiple degrees of freedom (DoF). Traditional control methods often involve complex computations and may not be sufficiently responsive for real-time surgical applications. Convolutional neural network (CNN)-based algorithms are developed to provide faster, more responsive control of a robotic arm with multiple DoF [46]. These algorithms can achieve E2E learning, directly mapping sensory inputs from image sensors to control commands without the need for intermediate processing steps. Compared to traditional control algorithms, the proposed CNN-based methods can handle the complexities of robotic control in highly dynamic and complex surgical settings. Traditional methods often require significant tuning and calibration to accommodate changes in the environment or the robot’s configuration. In contrast, CNNs can naturally adapt to variations, learning robust representations that generalise well across different scenarios. This adaptability reduces the need for manual adjustments and can improve the overall responsiveness and accuracy of the robotic system.

### Delay-robust compensation

Despite advancements in AI techniques for reducing delays by optimising network resources and improving data processing, some degree of delay in telesurgery and telementoring systems is inherently unavoidable due to physical and technical limitations. To address these intrinsic challenges, researchers have proposed AI algorithms that compensate for delays, enhancing system robustness and ensuring safe and effective surgical procedures.

One novel approach to compensating for visual and control information delays involves the use of a virtual reality (VR) simulation platform that provides the surgeon with a real-time virtual representation of the patient to the surgeon [44]. On the patient side, YOLOv3 and Mask R-CNN are employed to efficiently detect and track objects within the surgical scene, generating crucial pose information for the VR simulation. These networks are optimised for real-time processing, thereby reducing computational overhead. By transmitting only occasional object pose updates over the network, rather than continuous streams of large video frames, the system significantly reduces network resource consumption.

The surgeon’s actions are transmitted as high-level commands to the remote robotic system, which performs the surgical tasks on the patient. By breaking down surgical procedures into small, high-level instructions known as surgemes, the system transmits only these commands instead of full kinematic data streams. This further reduces network load and minimises latency. Although there are inherent processing delays of less than 1 s, the semi-autonomous framework allows the system to function effectively even with communication delays of up to 5 s. The platform is currently deployed on a desktop workstation due to the substantial computing power required, which is not yet feasible on wearable devices.

In addition to visual and control data compensation, the prediction of haptic feedback has also been explored to address delays in tactile sensations. Radial basis function (RBF) neural networks can be used to predict the start and stop times of haptic feedback signals [48]. While the study did not predict the detailed feedback signals, it could determine whether the remote surgical tool was interacting with a surface. The system operates on the remote surgeon’s side but requires sampling surface shape information from the patient’s environment. This surface data is used to train the RBF neural network, enabling it to predict feedback signal timings.

Error rectification on the patient side is another effective strategy to mitigate the effects of delays. This can be achieved through various means, including eliminating tremors in the surgeon’s movements, synchronising control signals between the surgeon’s console and the remote robot, and increasing the level of automation in the robotic system. For instance, one study [53] utilises long–short-term memory (LSTM), a type of recurrent neural network, to predict and automatically eliminate the surgeon’s physiological tremor. Traditional tremor suppression methods, such as low-pass filters, often introduce phase delays and reduced precision. The LSTM network is adept at handling non-stationary, time-series data, making it suitable for predicting tremor signals in real time. The study compared the LSTM approach with other methods like autoregressive (AR) and autoregressive integrated moving average (ARIMA) models, demonstrating a significant improvement in prediction accuracy and tremor suppression.

In another study, a delay-tolerant control strategy is developed to maintain accurate and synchronised control between the master console and the slave robot, even with delays of up to 600 ms [52]. The authors apply a barrier Lyapunov function (BLF) to constrain the synchronisation errors within predefined bounds. This mathematical approach ensures that the system operates safely, preventing overshoot and maintaining high precision despite communication delays.

Increasing the level of automation in surgical robots is also a viable method for compensating for delays, as it reduces the need for continuous remote control of delicate procedures. In [51], a portable stereotactic surgical robot was developed, featuring frameless registration, real-time tracking, and notably, semi-autonomous bedside intracranial catheter placement [55]. For tasks that are challenging to fully automate, semi-automatic motion prediction can provide sufficient lead time for actual control signals to arrive [56]. For example, the aforementioned VR platform [44] can recognise the surgemes the surgeon is performing and autonomously complete them if necessary. The platform is evaluated on a peg transfer task (a common robotic surgery challenge), resulting in an 87% task success rate, even with up to 5 s of delay.

Additional error-correcting features can be incorporated into the patient-side robot system to prevent errors caused by delay or other factors. Enabling robots to monitor their own movements in relation to the patient can prevent inadvertent injuries [45]. To achieve this, researchers designed and trained a conditional generative adversarial network (cGAN) model to process images from simulated gastrointestinal robotic surgeries from the 2015 EndoVis Instrument Challenge. The model infers the position of the robotic arms from visual data, effectively replacing traditional sensor-based systems and enhancing the robot’s situational awareness.

Similarly, in the context of deep anterior lamellar keratoplasty (a type of corneal surgery), accurate needle positioning is crucial. Researchers utilised U-Net powered real-time optical coherence tomography (OCT) image segmentation to guide the needle through the cornea [49]. The system focuses on separating the needle tip, corneal boundaries, and underlying tissue layers. It addresses common OCT image issues, such as refractive errors and optical noise. With segmentation errors as low as 7.4 $\mu$m for the upper corneal boundary and 3.6 $\mu$m for the needle tip, this approach enables automated and precise needle positioning.

## Discussion

A wide variety of AI models have been and are in development to address many of the technical challenges currently faced in telesurgery and telementoring. However, these solutions address a broad range of tasks and apply numerous model architectures [45, 49]. This limits the ability to combine various approaches to address the multifaceted limitations of remote surgery. Furthermore, most of the delay-robust applications are validated *in vitro* and *ex vivo*, rather than in a computer-simulated environment [44, 49, 51, 53].

There are unique strengths that AI-powered techniques present for the minimisation of delays or compensated effects of delays in telesurgery and telementoring. AI-based solutions can work with multimodal data and are more adaptable to different surgical scenarios, thus saving the time and effort to develop customised solutions for different data and surgical scenarios. Their strong modelling and predictive features on complex and large amounts of data are also well-suited for filling out the gaps caused by data loss, achieving more efficient data transfer, and avoiding surgical errors caused by delay and other factors. Solutions for coping with delays positively impact the broader dissemination of telesurgery and telementoring that help reduce travel costs, promote collaborative surgery, enhance surgical education, and allow fast emergency response.

Although we found only one study exploring AI-powered VR simulations specifically applied to telesurgery [44], we believe that such simulations hold immense potential for automated mentoring and training in remote surgical contexts. AI-powered VR simulations can provide immersive, hands-on training environments where surgical trainees can practice procedures with real-time feedback and guidance. Automated mentoring within these simulations could allow trainees to interact with complex surgical scenarios, refining their skills under varying degrees of difficulty, and receiving AI-driven insights that guide them toward best practices. This capability would be particularly valuable in underserved regions, where access to experienced mentors is limited, offering a scalable, accessible platform for surgical education. As wearable devices gain more computing power, VR simulation-based training can even be conducted with such devices instead of bulky robotic consoles, further reducing cost and boosting availability.

However, AI techniques bring an emerging number of limitations. Some delay compensation algorithms take a significant time to run. For instance, an AI-based surgical tooltip segmentation solution can have a processing delay of around 500 ms [49, 57]. Even a slightly faster solution has 299 ms delay [45]. The delays these solutions introduce are even greater than just transmitting the information using traditional approaches. Since the runtime of the AI solutions is an important concern, no work investigates the performance of specially designed efficient model architectures. The need for more translational research is more significant than ever before. When new technologies are applied to the clinical domain, patient and surgical team data security and privacy are of paramount concern. Since many AI-powered techniques are still considered black boxes, their outputs can not be reasoned. Also, many AI-powered techniques require training the models in a centralised manner, collecting data from multiple institutions. This approach greatly magnifies the security and privacy risks of patient data.

### Impact on surgical practice

Minimising latency in networking and processing constitutes a pivotal factor for efficient and effective telesurgical and telementoring experiences. Achieving negligible delays will enable remote surgeons to perform safe, efficient robotic surgery and deliver mentoring with a perceptual equivalence to being physically present in the same room as the patient. Further reduction becomes unattainable once network and processing delays have been optimised to their physical limits. For instance, the inherent constraint imposed by the speed of light necessitates a minimum signal propagation time of approximately 19 ms for communication from London (UK) to New York (US). In protracted long-distance scenarios, implementing delay compensation schemes becomes imperative to elevate the overall quality of surgical experiences and outcomes. AI assumes a crucial role in mitigating challenges arising from delays, offering multifaceted solutions ranging from video extrapolation to error rectification. However, whether these delay compensation schemes are safe for the patient is a significant concern. A few open questions still remain unanswered when evaluating the accuracy of compensation algorithms and their effect on remote telesurgery and telementoring. These concerns focus on the trustworthiness of these AI solutions. If possible, these solutions need to let surgeons differentiate real-time data from generated information. Obtaining this ability not only requires technology developers to come up with well-designed user interfaces to facilitate differentiation. It also requires special training for the surgical teams to understand the characteristics of the AI solutions. A standardised response must be specified and trained when the assistive automatic functions are used. Moreover, a vigilant monitoring team on the patient side should be able to intervene promptly in the event of errors in the actions of the robotic agent, regardless of the originating cause within the AI solution.

### Impact on surgical education

While AI has started to improve telementoring and telesurgery via the efficient allocation of resources, delay minimisation and algorithmic robustness, the efficient delivery of data to remote locations will also be impactful to surgical education. The Internet of Surgical Things has been one of the key technological advances for facilitating the capturing and delivery of heterogeneous data observed in the operating room. The seamless integration of different modalities and data from surgical devices adds value not only for telesurgery and telementoring but also for learning. This will extend beyond audiovisual material typically used during surgical training to being able to interpret other signals such as tactile information, data from an anaesthetic machine, or the interplay of the surgical team, among others. Through this approach, surgical trainees will be able to learn technical and non-technical skills holistically and contextually. While telesurgery and telementoring will allow trainees to learn from patient-specific cases in real time, heterogeneous data repositories in the cloud will allow trainees to learn from retrospective cases. Moreover, surgical data science will provide a tremendous amount of didactic content for surgical trainees, which is impossible to manually curate. AI models will be essential tools for surgical scene understanding, assessment of surgical skills, recognition of surgical workflows [58], granular identification of surgical actions, and surgical team dynamics, all in the context of patients and surgeons performing on them.

## Conclusion

Despite over two decades after the first surgical intervention was demonstrated remotely, today telesurgery and telementoring still face many challenges for a successful large-scale deployment. In this review, we report AI studies that address some of these challenges and contribute towards enhanced telesurgery and telementoring by optimising network resources, minimising processing delays, and providing delay-robust algorithms. These methods will have a positive impact on surgical practice and education to provide an experience that is truly holistic and contextual. Further collaborative research is needed to guarantee telesurgery and telementoring reach their full potential, at a global scale, for enhanced surgical practice and education.

## Data Availability

No data sets were generated or analysed during the current study.
